# Short-term exposure to unmanned aerial vehicles does not alter stress responses in breeding tree swallows

**DOI:** 10.1093/conphys/coaa080

**Published:** 2020-08-25

**Authors:** Bradley D Scholten, Abigail R Beard, Hyeryeong Choi, Dena M Baker, Margaret E Caulfield, Darren S Proppe

**Affiliations:** 1 Biology Department, Calvin University, Grand Rapids, MI 49546, USA; 2 Research Director, Wild Basin Creative Research Center, Saint Edward’s University, Austin, TX 78704, USA

**Keywords:** tree swallow, drone, stress, behavior, corticosterone, telomere

## Abstract

Recent studies suggest that visual and acoustic anthropogenic disturbances can cause physiological stress in animals. Human-induced stress may be particularly problematic for birds as new technologies, such as drones, increasingly invade their low-altitude air space. Although professional and recreational drone usage is increasing rapidly, there is little information on how drones affect avian behavior and physiology. We examined the effects of drone activity on behavior and physiology in adult, box-nesting tree swallows (*Tachycineta bicolor*). Specifically, we monitored bird behavior during drone flights and in response to a control object and measured telomere lengths and corticosterone levels as indicators of longer-term physiological stress. We predicted that drone-exposed tree swallows would habituate behaviorally after multiple flights, but that telomeres would shorten more quickly and that baseline corticosterone levels would be altered. One significant and two strong, non-significant trends in behavioral assays indicated that adult swallows acted more aggressively towards drone presence compared to a control object, but were slower to approach the drone initially. Swallows were also more reluctant to use nest boxes during drone activity. Tree swallows habituated to drone presence as expected, although the rate of habituation often did not differ between drone-exposed and control groups. Contrary to our prediction, drone activity did not affect telomere length, corticosterone levels, body mass or fledging rates. Overall, our results indicate that a small number of short, targeted, drone flights do not impact tree swallow health or productivity differently than a non-invasive control object. Minor behavioral differences suggest that increasing the frequency of drone use could impact this species. We provide some of the first results addressing how drone activity alters behavioral, physiological and molecular responses to stress in songbirds. A better understanding of these impacts will allow ecologists to make more informed decisions on the use and regulation of new drone technologies.

## Introduction

Natural landscapes are changing quickly, largely due to a growing human population and rapid urbanization. In addition to habitat loss, urbanization increases the exposure of wildlife to humans, roads, vehicles and their corresponding sounds (De Gregorio *et al.*, 2014; Haddad *et al.*, 2015; French *et al.*, 2017; Hunt and Vargas, 2018; Injaian *et al.*, 2018, 2019). Human development can impact ecosystems directly through processes such as habitat fragmentation and decreased species richness (McKinney, 2008; Haddad *et al.*, 2015). However, anthropogenic disturbance can also alter behavior and physiology in the animals that remain. For example, increased human exposure leads to decreased flight responses in sea turtles and changes in immune response and baseline corticosterone levels (CORT) in marine iguanas (French *et al.*, 2017; Hunt and Vargas, 2018). Heightened levels of boat traffic increase flight responses and diminish breeding performance in some seabirds (Marcella *et al.*, 2017; Monti *et al.*, 2018). Anthropogenic noise, a side-effect of human development, also alters physiology and behavior in animals (Wright *et al.*, 2007; Injaian *et al.*, 2018). Birds are particularly susceptible to noise, which can interfere with vocal communication (Curry *et al.*, 2018), decrease telomere lengths (Injaian *et al.*, 2019), reduce body condition and impact reproductive success (Wright *et al.*, 2007; Francis and Barber, 2013; Shannon *et al.*, 2016; Injaian *et al.*, 2018). Changes in baseline corticosterone levels have also been noted, although the direction of the effect has been variable. For example, in some cases, CORT has been found to increase (Injaian *et al.*, 2019), decrease (Kleist *et al.*, 2018) and not change (Flores *et al.*, 2019) in response to noise.

Small, remote-controlled unmanned aerial vehicles (UAVs or drones) represent another new source of anthropogenic disturbance that alters visual and acoustic activity in low lying airspaces (<120 m) typically inhabited by birds (Mulero-Pázmány *et al.*, 2017). Recreational drone use is increasing, and drones are also being used in biological research (Arts *et al.*, 2015; Lambertucci *et al.*, 2015; Lyons *et al.*, 2018; Mulero-Pázmány *et al.*, 2017; Scholten *et al.*, 2019). However, little is known about how drone use might impact wildlife (Lambertucci *et al.*, 2015). A handful of behavioral studies suggest that birds alter their state or location in response to drones, which likely indicates that some level of stress is occurring (Lyons *et al.*, 2018; Mulero-Pázmány *et al.*, 2017; Reintsma *et al.*, 2018). But, longer term measures of fitness impacts, via hormonal and molecular assays, are lacking.

The methodology for quantifying stress in free-ranging animals varies widely. Although many molecular studies report a single stress indicator (Lynn *et al.*, 2013; Dorado-Correa *et al.*, 2018), running multiple, complementary assays is a more robust method for quantifying stress (Tarlow and Blumstein, 2007). Two molecular protocols for measuring stress responses include quantification of glucocorticoid levels and telomere lengths (Horn *et al.*, 2008; Meillère *et al.*, 2015; Carneiro *et al.*, 2016; Quirici *et al.*, 2016; French *et al.*, 2017). In response to stress events, the hypothalamic–pituitary–adrenal axis is activated and CORT is released (Kleist *et al.*, 2018). Repeated stress events can cause a chronic stress response, ultimately changing baseline corticosterone levels, a measure of long-term fitness (Bonier *et al.*, 2011; Flores *et al.*, 2019). However, the amount and direction of change of baseline CORT is highly variable depending on individuals, species and circumstances (Bonier *et al.*, 2011; Curry *et al.*, 2018; Flores *et al.*, 2019; Injaian *et al*., 2019). Telomeres are repetitive, non-coding DNA sequences found on the ends of eukaryotic chromosomes that help keep the genome stable throughout the DNA replication process (Dorado-Correa *et al.*, 2018). They protect chromosomes during cell division and naturally shorten throughout the lifespan of an organism (Criscuolo *et al.*, 2009). Under stress, telomeres in birds (and other organisms) shorten at a higher rate (Meillère *et al.*, 2015). For this reason, telomere length is touted as a useful way to measure chronic stress in birds and likely in other animals as well (Kotrschal *et al.*, 2007; Monaghan, 2014; Meillère *et al.*, 2015; Quirici *et al.*, 2016). Collecting behavioral data alongside these molecular assays simultaneously enhances our ability to broadly quantify stress.

We aimed to quantify behavioral and molecular responses to drone use in a breeding songbird. Specifically, we tested the effects of aerial drones on physiological and behavioral stress responses in adult, box-nesting tree swallows (*Tachycineta bicolor*) during the breeding season. We hypothesized that repeated drone exposure would alter stress responses in tree swallows. We predicted that initial behavioral responses to drone activity would differ from responses to a benign control object, but that behavior might converge after several trials due to habituation [similar to Reintsma *et al.*, 2018 (birds); Ditmer *et al.*, 2019 (bears)]. Based on observed stress responses in previous studies, we also predicted that repeated drone flights would alter baseline CORT levels (Bonier *et al.*, 2011; Kleist *et al.*, 2018) and that telomere length would shorten at a higher rate compared to a control group (Dorado-Correa *et al.*, 2018; Quirici *et al.*, 2016).

## Methods

### Species and site selection

All methods were reviewed and approved by the Calvin University Institutional Animal Care and Use Committee (Protocol: BR2018-01). Tree swallows nest readily in boxes and are relatively easy to monitor and capture for blood collection (Injaian *et al.*, 2019)—making them an ideal species for research on avian stress in response to repeated drone flights. We studied two tree swallow populations located in western Michigan, USA. The first site was located at Grand Valley State University in Ottawa County (Supplementary Fig. S1; 42°57’33”N 85°53’53”W). This 14.5 acre site contained 101 nest boxes in a rural setting that included several small ponds. Boxes used in the study were separated by >15 m. Recreational drone use was not allowed at this site, and other sources of acoustic and visual disturbance were minimal. The second location was Egypt Valley Country Club in Kent County (43°00’41”N 85°29’53”W). Other than short periods of mowing, this site was also relatively quiet and was off-limits to recreational drone use. Over 60 paired boxes were separated by >20 m across this ~320 acre course. Golf-related events limited access to the site, resulting in lower sample sizes at this location.

### Field protocol and behavioral assays

Fifty-two pairs of nesting tree swallows were monitored from May 28 to June 28, 2019. Twenty-six pairs of breeding tree swallows were exposed to drone activity and assessed for behavioral stress. Twenty-six additional tree swallow pairs were not exposed to drone activity, but received similar stress assessments (i.e. control). Molecular responses to stress were also assessed for a subset of 22 individuals from each treatment group (similar to Curry *et al.*, 2018, Injaian *et al.*, 2018, 2019). Research activities began at each box after all eggs hatched and ended before young fledged. This timeframe was chosen because adults are instinctually driven to attend to their nestlings regardless of disturbance level, ensuring that target adults were present during all drone and control treatments. At least one day prior to initiating drone or control activity, the nesting female (and occasionally the paired male) was captured and banded with a metal US Fish and Wildlife Service band and one color band for easy identification. Birds were captured using a one way flap trap placed over the box opening or by manually covering the box opening while the adult was inside (Garlick *et al.*, 2014). Once in hand, we measured wing, tail, bill and tarsus length to the nearest 0.1 cm and determined the mass of each bird to the nearest 0.1 g using a digital scale (AWS-600, Atlanta, Georgia, USA). One to three days after drone or control trials were complete, each bird was captured a second time to collect post-experimental mass and a second blood sample.

Blood samples were taken from the brachial vein (Owen, 2011) within 3 minutes of capture to quantify baseline CORT levels before elevation occurred due to handling (Curry *et al.*, 2018). Up to 150 μl of blood was collected with one or two 75 μl heparinized capillary tubes and placed on ice for up to 5 hours before processing. One drop of blood was placed on an FTA card (GE Life Sciences, Pittsburgh, Pennsylvania, USA) for DNA extraction and telomere analysis (Quirici *et al.*, 2016). DNA was extracted from FTA cards using the ‘DNA Extraction and Purification of Dried Blood’ protocol from the E.Z.N.A. Blood DNA Mini Kit (Omega Bio-tek, Inc., Norcross, Georgia, USA).

We used a DJI Inspire 1 (DJI North America, Los Angeles, California, USA) for all drone flights. The drone was launched from the ground at 1.5–3 m from the target nest box and then positioned immediately at 1.5 m above the center of the nest box. Each flight lasted for a total of 6 minutes, with flight patterns alternating between 1 minute blocks in the hover position and horizontal movement within 10 m of the box in an X and diamond pattern. The control condition was designed to provide a discrete event with an object similar in size and coloration (white) to the drone, but without noise or movement. We placed a 60 cm section of 5 cm polyvinyl chloride (PVC) pipe mounted horizontally on a 1.5 m green pole (2.5 cm in diameter) < 1 m away from the target box. The control remained stationary for the entire 6-minute trial. Four trials were repeated at each box within a 10-day period for both drone and control treatments. Prior to initiation of each trial, we approached the box to make sure the bird was not in the box. In most instances, adult presence near the box was confirmed visually at this time. Caution was taken to not treat boxes in close proximity on the same day in order to avoid secondary exposure. To reduce the likelihood that birds using control boxes were exposed to drone activity, and vice versa, boxes in different treatments were spatially or temporally segregated (i.e. a drone box may have been next to a control box from a previous round; Supplementary Fig. S1). During all trials, two observers quantified behavior from a position >30 m from the box location.

Behavioral assays included counting the total number of tree swallows responding to the treatment (i.e. maximum number of birds within 15 m of the object simultaneously), as well as the average number of swoops (e.g. aggressively mobbing or diving; Winkler, 1992; Sharman *et al.*, 1994) directed towards each object per swallow responding (calculated as the mean number of swoops per bird). The number of times an adult swallow landed on or entered the box during a trial was also counted. Although multiple swallow pairs responded aerially to treatments, box attendance was generally limited to the target breeding pair. We also recorded latency (in seconds) to the first approach within 3 m of the drone or control object, latency to enter the target box once a trial was initiated and latency to enter the box after trial completion. The latter measure was capped at 2 minutes. Total time spent within the box during each trial was also tabulated. Finally, nestling fate was monitored at the Grand Valley State University site twice weekly until the day of fledge to determine whether reproductive success was impacted by drone exposure.

### Molecular stress response assays

Within 5 hours of collection, blood samples were transferred from capillary tubes to 0.5 ml Eppendorf tubes. Blood was then centrifuged for 5 minutes at 10 000 × g, deemed complete when there was clear separation of plasma from red blood cells. Red blood cells and plasma were stored separately at −80°C until used for assays. CORT levels were measured using an enzyme immunoassay kit (ELISA) from Enzo Life Sciences (Corticosterone ELISA Kit; Owen, 2011; Lynn *et al.*, 2013; Lynn and Kern, 2014). Samples were performed in duplicate. Results were read by an Eon Microplate Spectrophotometer (BioTek Instruments, Winooski, VT, USA) and then converted to CORT levels in ng/ml using the manufacturer’s instructions.

Telomere lengths were quantified using quantitative polymerase chain reaction (qPCR) with glyceraldehyde-3-phosphate dehydrogenase (GAPDH) serving as the single control gene as previously described with minor modifications (Criscuolo *et al.*, 2009; Meillère *et al.*, 2015; Dorado-Correa *et al.*, 2018). The following GAPDH primers (optimized for the American redstart; *Setophaga ruticilla*) were used due to a reduced level of primer dimers based on qPCR melting-curve results as compared to published zebra finch (*Taeniopygia guttata)* sequences (Criscuolo *et al.*, 2009). Primer sequences were 5′ to 3′ Forward: TGACCACTGTCCATGCCATCAC and Reverse: TCCAGACGGCAGGTCAGGTC (Quirici *et al.*, 2016). The amplification efficiencies for GAPDH and telomere amplification were within the accepted range of 100 +/ − 15%. The 20 ng DNA samples from each individual bird were run in triplicate, Ct values were averaged and quantified based on a plate-specific standard curve and a pooled sample to serve as a reference sample to account for interplate variability. The telomere to single gene (T/S) ratio was calculated based on the method described in Criscuolo *et al.*, (2009). Reichert *et al.* (2017) noted that extraction from FTA cards can result in shorter telomere lengths compared to other methods. However, FTA storage periods were relatively short (<20 days), and techniques were standardized across all samples. Thus, any bias towards shortened telomeres should be uniform across groups and is unlikely to confound our comparative results.

### Data analysis

Behavioral data were compared using generalized linear mixed effects models (glmer, package lme4, R version 3.3.3; Bates *et al.*, 2015; R Core Team, 2019) in program R. Dependent variables included: number of tree swallows responding, number of swoops per bird, number of box lands, number of box entrances, and total time spent in the box. Independent variables for these behavioral models were treatment (drone/control), trial number (1–4), the interaction between treatment and trial number, date (Julian), time of day (minutes since midnight), hatch date (JulianHatch), brood size (NumHatch) and recorder ID. Box ID and location were included as random effects.

Survivorship-style logistic regression (Hazler, 2004; Valentine *et al.*, 2019) was used to analyze three additional dependent terms: latency of first approach to within 3 m of the object (drone or control), latency to enter the box during the trial and latency to enter the box post-trial. Each dependent term included a binomial numerator [event did/did not occur] and an integer denominator [time (s) until event occurred]. Thus, latency analyses reported the likelihood that an event (e.g. box entrance) occurred within a unit of time (per second, since this was the unit of measure). Independent variables for logistic models were treatment (drone/control), trial number (1–4), the interaction between treatment and trial number, date (Julian), time of day (minutes since midnight), hatch date (JulianHatch), brood size (NumHatch) and recorder ID, with box ID and location included as random effects.

Pre- and post-experimental telomere lengths (T/S ratios), mass and CORT levels were analyzed using separate generalized linear mixed effects models. The independent variables of interest were treatment (drone/control), sampling period (preliminary/post) and the interaction between these two terms. Date (Julian), time of day (minutes since midnight), brood size (NumHatch), and the number of days after the last trial that the post-trial blood sample was taken (DaysAfter) were also included to control for potential variance related to these terms. Band number, sex and location were included as random effects. Fledging rates for control and drone broods were compared using a two sample *t*-test.

**Figure 1:**
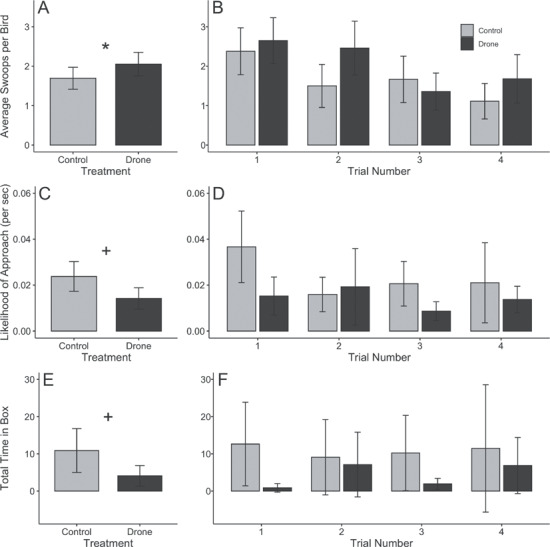
Behavioral trends between treatments. A significant difference in behavior between drone and control trials was observed for (**A**) average swoops per bird [(**B**) treatments split by trial]. Strong (*P* < 0.10), but non-significant, trends were observed for (**C**) likelihood of approaching the object [per unit time (s)] during drone and control trials [(**D**) treatments split by trial] and (**E**) total time in box during the trial [(**F**) treatments split by trial]. Means and confidence intervals are derived from raw data. ^*^ = *P* < 0.05, + = 0.05 < *P* < 0.10.

**Figure 2:**
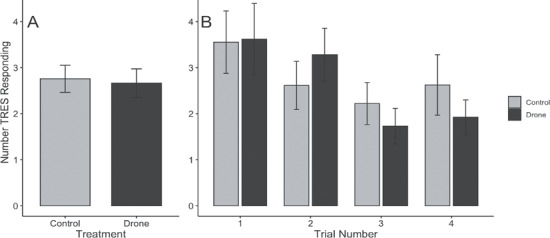
Number of tree swallows responding. (**A**) Treatment did not differ, but there was a significant difference in (**B**) trial number, with fewer birds responding to both objects in subsequent trials.

**Figure 3:**
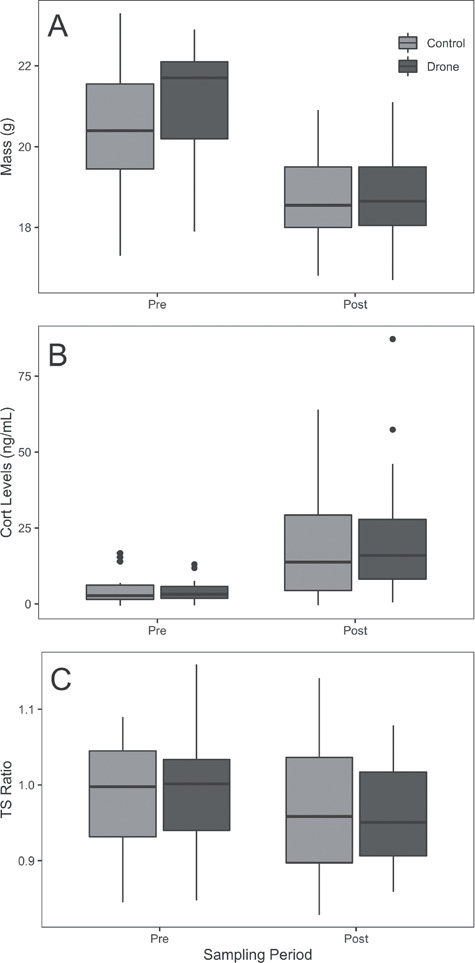
Physiological data. (**A**) Change in mass: neither sampling period nor treatment had significant differences. (**B**) Change in baseline corticosterone: no significant difference between sample or treatment in baseline CORT levels. To increase visual acuity, we removed one outlier in the post experimental control group at 124.27 ng/ml. (**C**) Change in relative telomere length: no significant difference between sample or treatment in the rate of telomere shortening.

## Results

### Behavioral data

One behavioral measure was significant at α = 0.05, and, in two cases, a strong but statistically non-significant trend was present (*P < 0.10*; Supplementary Table S1). When tree swallows responded to drone exposure, they swooped at the object significantly more often than towards a control object (*X ^2^* = 3.91, df = 1, 200, *P* = 0.048; Fig. 1A and B). During drone trials, the likelihood of object approach was generally lower (*X ^2^* = 3.17, df = 1, 206, *P* = 0.075; Fig. 1C and D), and birds spent less time inside the box (*X ^2^* = 3.14, df = 1, 207, *P* = 0.076; Fig. 1E and F), though both patterns were non-significant. Behavior did not differ between treatments for the other five terms: number of tree swallows responding (*X ^2^* = 0.15, df = 1, 204, *P* = 0.696; Fig. 2), number of box lands (*X ^2^* = 1.46, df = 1, 207, *p* = 0.226; Supplementary Fig. S2), number of box entrances (*X ^2^* = 0.01, df = 1, 207, *P* = 0.904; Supplementary Fig. S3), likelihood to enter the box during the trial (*X ^2^* = 0.02, df = 1, 206, *P* = 0.887; Supplementary Fig. S4) and likelihood to enter the box after the trial (*X ^2^* = 2.22, df = 1, 207, *P* = 0.136; Supplementary Fig. S5). As predicted, swallows habituated as trials proceeded, with significantly fewer birds responding to both control and drone events (*X ^2^* = 15.56, df = 3, 202, *P* < 0.001; Fig. 2). Although not significant for other behavioral measures (*X ^2^* < 4.90, *P* > 0.179), a similar trend is visible with the average number of swoops per bird (Fig. 1A and B), number of box lands (Supplementary Fig. S2), number of box entrances (Supplementary Fig. S3) and the likelihood of box entrance during the trial (Supplementary Fig. S4).

### Physiological and fledging data

A small number of plasma samples could not be analyzed post-experiment, resulting in a reduced sample size of 19 individuals per group for CORT comparisons. In a few cases, birds escaped before assessing mass, resulting in 19 available individuals per group for treatment comparisons. No significant differences were found between treatment groups prior to drone or control exposure for baseline corticosterone (Welch’s *t* = 0.43, df = 32.14, *P* = 0.668) or telomere lengths (Welch’s t = −0.10, df = 43, *P* = 0.924; Supplementary Table S2). Neither treatment nor the interaction between treatment and sampling period (preliminary/post) were significant for mass (Treatment: *X ^2^* = 3.48, df = 1, 88, *P* = 0.062; Treatment*Sample: *X ^2^* = 1.88, df = 1, 88*, P =* 0.171*;*Fig. 3A), corticosterone levels (Treatment: *X ^2^* = 0.83, df = 1, 78, *P* = 0.364; Treatment*Sample: *X ^2^* = 0.58, df = 1, 78, *P =* 0.447*;*Fig. 3B) or telomere lengths (Treatment: *X ^2^* = 0.004, df = 1, 89, *P* = 0.949; Treatment*Sample: *X ^2^* = 0.24, df = 1, 89*, P =* 0.624; Fig. 3C), indicating that any impact of drone exposure did not differ from the addition of a non-invasive, control object. Although not significant, a temporal trend towards decreased mass (*X ^2^* = 2.51, df = 1, 88, *P* = 0.113; Fig. 3A) and increased CORT levels (*X ^2^* = 0.18, df = 1, 78, *P* = 0.674; Fig. 3B) is also visible. Fledging rate from boxes that were not predated was 81.6% for drone-exposed pairs (*n* = 21 boxes, 95 young) and 80.1% for control pairs (*n* = 21 boxes, 93 young), with no significant difference between treatment groups (*t* = 0.17, df = 36, *P* = 0.865).

## Discussion

Tree swallows altered several behaviors during drone exposure in a pattern that suggests perceived object intrusion and box aversion, although most individual trends were not strong enough to reach significance. Specifically, swallows were less likely to approach a drone than the control object, but displayed more aggressive behavior towards the drone—indicated by a significantly greater number of swoops per bird responding. In addition, swallows spent comparatively less time in the box during drone flights compared to the control object. It is possible that tree swallows perceived the drone as a threat and minimized time in their box during trials to enhance physical safety (Wheelwright and Dorsey, 1991). It is also plausible that the turbulence created by the drone rotors reduced a bird’s physical ability to fly into their box, necessitating increased avoidance of the area. Due to physical limitations, the latter explanation would also predict fewer box visits, a pattern that was not observed. If a drone is perceived as a threat, box avoidance may be balanced with the need to care for young. In this case, birds might visit boxes just as often to feed nestlings, but vacate the area more quickly to reduce risk.

It was also evident that tree swallows habituated to the presence of a drone over time. However, in many cases the pattern was no different than habituation observed in the control group (e.g. number of box entrances). Tree swallows are an exploratory species and often react strongly to a novel object in their territory (Winkler, 1992; Sharman *et al.*, 1994). Thus, it is not surprising that drone and control objects both created an initial increase in activity, which subsided over time. However, the relative similarity in response patterns between drone and control objects suggests that the drone may not be perceived as a high level threat in this species.

The mild or absent impact of short-term drone exposure in breeding tree swallows is also supported by the lack of a comparative effect on corticosterone levels, mass or telomere lengths. The lack of physiological differences indicates that there were no markers of chronic stress as a result of drone exposure that exceeded what could result from a non-invasive control object. Mass and corticosterone levels changed between pre- and post-experimental samples, although without significance. These patterns are likely due to the stress associated with rearing young (Angelier and Chastel, 2009). The presence of natural breeding stress might also partially explain why drone activity had so little perceivable impact on exposed swallows. However, breeding is also a timeframe when even minor stressful events can result in lower reproductive success (Francis and Barber, 2013; Shannon *et al.*, 2016; Injaian *et al.*, 2018). Notably, fledging success was not affected, again suggesting that drone use had little impact on tree swallows.

It could be argued that our drone flights were too short or too sparse to impact tree swallow stress at the molecular level. However, our experimental design mimics the types of exposure that birds experience currently and likely for the foreseeable future. Drone battery life is limited to small units of time (often <20 minutes) and recreational users tend to explore large spatial areas rather than focus on a particular target. Even most scientific studies are unlikely to spend more time over a particular breeding pair than the durations used for our study. Nonetheless, reduced box attendance indicates that longer, more frequent flights could have an impact that was not observable in our study. As plans for commercial drone delivery services (e.g. Amazon Prime Air) that focus on regular use of particular routes advance, longer-term studies might become beneficial. Other variations of this study such as using different types of drones (e.g., larger or louder) could also be worthwhile.

In our study, a relatively inert, non-invasive object served as a control rather than a completely non-manipulated nest box. This technique was chosen because assessing behaviors such as latency to approach or number of birds responding at a non-manipulated nest box in the absence of an event becomes rather difficult to compare to an event, such as a drone flight. However, the lack of completely unmanipulated boxes also necessitates that any changes as a result of drone activity must be compared to the control object, rather than to an environment completely lacking intrusion. It is theoretically plausible that both the control and drone object significantly altered behavioral and molecular markers of stress. But placement of a silent, non-mobile object seems unlikely to produce this type of impact given that most box-nesting tree swallow populations also experience territory intrusion from conspecific and heterospecific birds species, small mammals, incidental human foot traffic and weekly nest checks. Further, tree swallows are a gregarious species, known for accommodating other types of human disturbance (Winkler *et al.*, 2004). Due to this latter trait, we caution drone users and developers to not over-extrapolate our non-significant results. More sensitive box-nesting species like eastern bluebirds (*Sialia sialis*) or open-nesting species like red-winged blackbirds (*Agelaius phoeniceus*) may be more strongly impacted by drone activity. Further, drone use may have a stronger impact on health and habitat use during more mobile life stages, such as migration.

Here, we report that four, 6-minute flights over a 10-day period had little to no more of an effect on tree swallow behavior, condition, physiology, or reproductive success than a non-invasive control object. For most of the behaviors assessed, tree swallows were initially wary of both drone and control objects, but habituated with additional trials. However, small reductions in box attendance during drone flights points towards potential impacts if the frequency and duration of drone usage near bird nesting sites increases in the future. We recommend that caution be used when extrapolating our results beyond tree swallows because sensitivity to noise and motion varies widely between species (Francis and Barber, 2013; Shannon *et al.*, 2016). Nonetheless, we provide the first quantitative behavioral and molecular investigation of drone impacts on stress in a native songbird species. Our results suggest that in tree swallows, nesting adults are more resilient to drone activity than might be expected. If appropriate caution and evaluation is in place, drone use may be a viable, non-invasive research tool for field investigations into songbird demographics and behavior.

## Funding

This work was supported by the Arnold and Mabel Beckman Foundation through the Beckman Scholars Program and the Calvin University Science Division.

## Competing Interests

The authors declare no competing interests.

## Data availability

Raw data and code are available at: https://osf.io/5fq49/?view_only=cd0dae910ef0482e93d57cd3fdc83984. 
